# Underground solution to the long-time solar fusion reaction puzzle

**DOI:** 10.1093/nsr/nwaf272

**Published:** 2025-07-12

**Authors:** Yuchen Jiang, Weiping Liu

**Affiliations:** China Institute of Atomic Energy, China; Department of Physics, Southern University of Science and Technology, China; China Institute of Atomic Energy, China; Jinping deep-underground Frontier Science and Dark Matter Key Laboratory of Sichuan Province, China

## Abstract

A promising experiment is proposed, which aims to resolve the long-standing solar metallicity puzzle by reducing the uncertainty from microscopic nuclear reactions at the deepest underground laboratory.

It has been nearly a year since the passing of Mr. Tsung-Dao Lee, a pioneer who made remarkable contributions to the scientific community. During his doctoral research, inspired by Fermi and under Chandrasekhar’s guidance, he investigated key nuclear reactions inside white dwarfs, including $^{3}$He + $^{4}$He (also called the $\alpha$ particle) fusion [1]. His vision of precise measurements for these crucial reactions more than 70 years ago still remains relevant and instructive today, particularly for the $^{3}$He($\alpha$, $\gamma )^{7}$Be reaction crucial for resolving the solar metallicity problem, and will be possible deep underground.

The Sun, our closest star, serves as a fundamental laboratory for studying astrophysics and cosmology. Despite advances in the standard solar model [2] since the recognition of nuclear fusion’s role in the evolution of the Sun over a century ago, significant challenges persist, notably the solar metallicity puzzle. This puzzle manifests as an $\sim\! 30\%$ discrepancy in the abundance of metals determined from helioseismology and solar spectroscopy, leading to the competing high-metallicity (high-*Z*) and low-metallicity (low-*Z*) solar models [3,4].

Recent progress in multi-messenger astronomy, particularly the solar neutrino flux measurements, provides new insights. The flux measurement of neutrinos from the proton-proton (pp) chain (see Fig. 1a), especially that of $^{7}$Be neutrinos with uncertainties as low as 2% [5], has emerged as a new tool for investigating solar metallicity, i.e. using neutrino flux predictions to put a stronger constraint on solar models.

**Figure 1.**
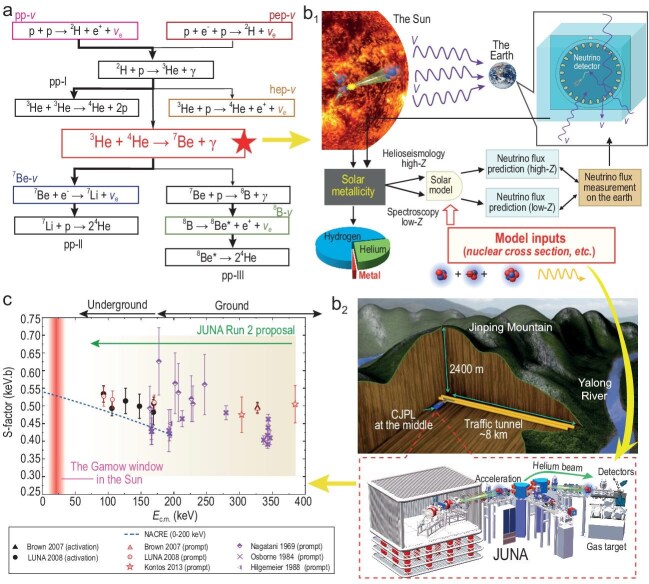
The JUNA proposal of using a high-precision measurement of the $^{3}$He($\alpha$, $\gamma )^{7}$Be reaction cross section to study the solar metallicity. (a) The nuclear fusion pp chain in the solar interior. Note that the line widths represent branching ratios, with thicker lines indicating stronger branches. (b.1) A simplified schematic diagram of how the nuclear reaction cross section constrains the solar metallicity. (b.2) A schematic view of the China Jinping Underground Laboratory and the JUNA platform. (c) Current status and the JUNA Run 2 proposal of the measurement of the $^{3}$He($\alpha$, $\gamma )^{7}$Be reaction cross section. Note that in panel (c) the NACRE recommended trend is represented by a blue dashed line, the prompt $\gamma$-ray measurements are represented by red (modern data) or magenta (old data) points, the $^{7}$Be decay measurements (activation data) are represented by black points and the energy coverage range of the JUNA Run 2 proposal is represented by a light yellow band and a green arrow.

One of the most significant inputs of modern solar models is the cross section of nuclear reactions (or the so-called astrophysical *S* factor), especially for predicting solar neutrino fluxes. The

largest uncertainty in predicting the $^{7}$Be neutrino flux stems from the cross section of the $^{3}$He($\alpha$, $\gamma )^{7}$Be reaction [2], which marks the onset of the pp-II and pp-III chains. Measuring this reaction cross section with a precision of 2%–3% would allow us to resolve the high-*Z* versus low-*Z* solar model debate as the measured solar $^{7}$Be neutrino flux provides a strong constraint to the models [6] (see Fig. 1b.1).

However, direct measurement of the $^{3}$He($\alpha$, $\gamma )^{7}$Be reaction cross section is extremely challenging. The significant environmental background interference resulting from the cosmic ray substantially limits further extension of the measurable energy range to the crucial Gamow region in laboratories on the Earth’s surface. Fortunately, the establishment of underground laboratories with ultra-low background levels has provided new opportunities. To date, the LUNA collaboration in Italy has pushed the measurement of the $^{3}$He($\alpha$, $\gamma )^{7}$Be reaction cross section to the lowest-energy level [7]. Nevertheless, due to the limitations of the LUNA accelerator energy, only measurement up to $E\mathrm{_{c.m.}} = 170$ keV was achieved, creating a gap with modern ground-based measurements at $E\mathrm{_{c.m.}}\ge 300$ keV. It has been demonstrated that the cross-section trend and characteristic resonance structure at higher-energy regimes (e.g. the resonance near $E\mathrm{_{c.m.}} = 3000$ keV [8]) play a critical role in our understanding of physical phenomena at lower energies (e.g. *R*-matrix parametrization [9]), revealing the importance of high-precision data in the medium-to-high energy range. Taking multiple datasets into account, despite significant contributions such as those made by the LUNA collaboration, it remains insufficient to resolve the solar metallicity puzzle.

Therefore, there is an urgent need for high-precision measurements of the $^{3}$He($\alpha$, $\gamma )^{7}$Be reaction cross section, especially in the energy range as close as possible to the solar Gamow region (for details of the Gamow region, see, e.g. [10]). However, it remains a great challenge because of the reaction’s extremely low cross section. For instance, under solar core conditions at a temperature of 16 MK, the Gamow peak lies around 25 keV (see Fig. 1c), where the cross section is estimated to be $\sim\! 10^{-18}$ barn—several orders of magnitude lower than the current detection limit achievable by cross-section measurements, especially at the ground level.

The Jinping Underground Nuclear Astrophysics (JUNA) collaboration [11] has proposed to measure the $^{3}$He($\alpha$, $\gamma )^{7}$Be reaction cross section within an energy range of $E\mathrm{_{c.m.}} = 80$–380 keV (see Fig. 1c) using both online prompt $\gamma$-ray and offline decay detection techniques, with major support from the National Natural Science Foundation of China (NSFC). The China Jinping Underground Laboratory (CJPL), located within a traffic tunnel of the Jinping hydropower project and shielded by 2400 m of overburden, is the world’s deepest underground laboratory with dramatically reduced cosmic ray background interference [12] (see Fig. 1b.2). Benefiting from the exceptional low-background environment provided by CJPL, combined with the advanced high-intensity He$^{1+,2+}$ ion sources and the accelerator system developed by the JUNA collaboration [13], the JUNA Run 1 experiments have released many interesting nuclear astrophysics research results [14]. Thanks to the excellent experimental conditions at the JUNA platform, the accessible energy range for $^{3}$He($\alpha$, $\gamma )^{7}$Be cross-section measurements is anticipated to extend significantly closer to the solar Gamow window and bridge the gap in the aforementioned 170–300-keV energy range. This will significantly improve the precision and accuracy of the data and impose the strongest constraint on contemporary solar models from the perspective of nuclear physics.

In the JUNA Run 2 [13] proposal, He$^+$ and He$^{2+}$ beams will be used with typical intensities of 2 and 0.5 pmA, respectively. A windowless gas target thickness of $10^{17}$–$10^{18}$ cm$^{-2}$ and a background level deduced from previous data in JUNA Run 1 were assumed. Under such conditions and at the lowest energy of 80 keV, the signal-to-noise ratio to measure the direct capture $\gamma$ ray to the ground state of $^7$Be when using a high-purity germanium detector placed at $90^{\circ }$ and shielded by a bismuth germanium oxide anti-coincidence array as well as high-purity oxygen-free copper and lead of appropriate thickness from the inside out is expected to exceed 5, allowing one to reach a statistical uncertainty of less than 3.5% in under 300 h of data collection. With extended data acquisition, the statistical uncertainty will be further reduced to below 2%.

For measurements at higher-energy points (e.g. $E_{\mathrm{c.m.}}\ge 170$ keV), the significantly larger cross section allows us to achieve a statistical uncertainty of less than 1%, facilitating a seamless data integration, which has not been achieved previously. Furthermore, provided that the cross section is sufficiently large, the deployment of multiple detectors will enable, for the first time, measurements of the angular distributions for direct captures to both the ground state and the first excited state, which have not yet been explored within such a low-energy range and represent the dominant source of systematic uncertainty in the LUNA measurements. Consequently, the systematic uncertainty arising from the angular distributions will be substantially reduced.

In summary, with strong support from CJPL and NSFC, the JUNA collaboration has proposed and will conduct high-precision measurement of the $^{3}$He($\alpha$, $\gamma )^{7}$Be reaction within the energy range closest to the solar Gamow region, thus imposing the strongest constraint on contemporary solar models. This will potentially enable us to touch upon the solution to the solar metallicity puzzle. We sincerely hope that the seven-decade-old vision of Mr. Tsung-Dao Lee will be fulfilled in the deepest underground laboratory characterized with ultra-low background conditions and unparalleled beam intensities for nuclear astrophysics experiments, i.e. the JUNA platform, in the near future.
